# Factors Affecting Communication Patterns between Oncology Staff and Family Members of Deceased Patients: A Cross-Sectional Study

**DOI:** 10.1371/journal.pone.0162813

**Published:** 2016-09-28

**Authors:** Tal Granot, Noa Gordon, Shlomit Perry, Shulamith Rizel, Salomon M. Stemmer

**Affiliations:** 1 Davidoff Center, Rabin Medical Center, Petah Tikva, Israel; 2 Sackler School of Medicine, Tel Aviv University, Tel Aviv, Israel; University of Stirling, UNITED KINGDOM

## Abstract

**Objective:**

Perceptions of the role of oncology medical staff in supporting bereaved families have evolved with the transition to interdisciplinary cancer care. We investigated the interactions between oncology professionals and bereaved families.

**Methods:**

This cross-sectional study involved all oncology medical staff at the Davidoff Center. Participants were given a questionnaire relating to bereavement follow-up. Responses were measured using a 5-point Likert scale.

**Results:**

Of 155 staff members, 107 filled questionnaires with <20% missing data and were included in the analysis (α = 0.799; corrected, α = 0.821). Respondents included physicians (35%), nurses (46%), social workers (7%), psychologists (4%), or unspecified (8%); 85% were Jewish, and 60% had ≥10 years of oncology experience. Most respondents thought that contacting bereaved families was important (73%), and that it provided closure for staff (79%); 41% indicated that they contacted >50% of the families of their deceased patients. Contacting bereaved families was considered the responsibility of the physicians (90%), nurses (84%), or social workers (89%). The main barriers to contacting bereaved families were emotional overload (68%) and lack of time (63%); 60% indicated a need for additional communication tools for bereavement follow-up. In a multivariate analysis, profession (physician vs. nurse), primary workplace (outpatient setting vs. other), and self-defined religion were significant variables with respect to the perceived importance of contacting bereaved families and to actually contacting them. Other factors (e.g., age, gender) were non-significant.

**Conclusions:**

Perspectives regarding bereavement actions differ significantly across medical professions, work settings, and self-defined religions. Additional guidance and education regarding bereavement actions is warranted.

## Background

Health professionals caring for cancer patients often deal with the death of patients who succumb to their illness. Family members of deceased patients often seek information and support from oncology staff, even if only for one last conversation [1–3]. Studies have revealed a gap between families of deceased patients' desire to have some interaction with the oncology staff who cared for their loved ones, and the occurrence of such interactions (73% of families would like an interaction with a team member, whereas such an interaction only took place in 22% of cases) [4]. The medical literature addressing end-of-life issues discusses the responsibility of medical staff to the families of dying cancer patients and debates whether this responsibility continues beyond the patients’ death, that is, whether medical staff should support grieving families (support that may also help the staff members gain closure regarding their patients’ death). In particular, the medical literature addresses the following questions: i) Is contacting family members of deceased patients within the duties of oncology professionals? ii) If so, who should be the one to reach out (e.g., medical oncologists, oncology nurses)? iii) What are the common communication patterns between oncology professionals and bereaved families, and are some preferable to others? And iv) What are the factors affecting the willingness of oncology professionals to contact bereaved families?

Notably, most of the studies addressing this question are outdated and refer only to physicians; however, two more recent studies are noteworthy [5, 6]. These studies surveyed 700–800 physicians (response rate, 20–70%) and found that 33–70% of the respondents reached out to bereaved families as part of their routine practice. Of the various disciplines examined, palliative care specialists reported the highest rate of contacting bereaved families. One of these studies also found that female sex, working in an academic setting, and palliative care specialty, were all associated with higher rates of contacting bereaved families. whereas, lack of time, no formal palliative care program, and not knowing which family member to approach were associated with lower rates of contacting bereaved families. Some of the respondents indicated that they lacked tools to deal with such interactions [5]. Despite the limited and/or contradicting data available regarding patterns of interaction between oncology staff members and bereaved families, the issue remains on the professional agenda, along with the underlying understanding that bereavement follow-up is important [5, 7, 8].

With the development of an interdisciplinary cancer care approach at the Davidoff cancer center, including physicians, palliative care members, nurses, and social workers, we thought that the issue of bereavement patterns should be further investigated. Thus, our objectives were to evaluate the importance attributed to reaching out to bereaved families by various oncology professionals, to explore whether staff members consider this outreach to be within their professional duties, to characterize the communication patterns between oncology staff and bereaved families, and to identify the factors preventing them from contacting these families.

## Methods

This was a single center cross-sectional study. The study was approved by the ethics committee of Rabin Medical Center.

### Research tools

A questionnaire was prepared specifically for this study by the researchers (Factors Affecting Communication Patterns Questionnaire) (S1 File). It included a total of 39 statements (20 statements, of which 6 included sub-statements). The statements referred to: i) the caregiver’s attitude towards communication patterns with bereaved families; ii) the type/frequency of interactions with the bereaved families; and iii) barriers and needs for additional tools. Responses were measured using a 5-point Likert scale (1, disagree; 5, agree), and participants could add free-text responses for each statement. A second questionnaire collected information on participants’—characteristics and work experience, including age, gender, country of birth, religion, marital status, number of children, discipline, position, years of oncology experience, work setting, and number of terminally-ill cancer patients treated each month.

In view of the contradicting findings among studies, and given Israel's multicultural society, we thought that additional factors such as socio- demographic, culture, age, marital status and country of birth may affect behavior.

### Participants

Study participants consisted of the 155 staff members of the Davidoff Center for Oncology and Hemato-Oncology, including physicians (35 and 26, respectively), nurses (45 and 35, respectively), psychologists (4 in total) and social workers (10 in total).

### Procedures

Researchers distributed the questionnaires to staff at the Davidoff Center, and collected the completed ones. All Davidoff health care providers were approached, and the study was presented at regular meetings. In order to ensure respondents' anonymity, they were instructed to put their responses in empty envelopes placed at each collection point. Questionnaire collection took approximately 6 weeks.

### Statistics

Cronbach's alpha coefficient was used to evaluate the questionnaires' internal consistency. A Chi-squared test was used to assess differences between groups (*p*<0.05 was considered significant). Multivariate analysis was used to identify significant variables associated with bereavement follow-up (covariates were: age, gender, country of birth, marital status, number of children, years of oncology experience, number of dying patients treated by the oncology professional, and work setting).

## Results

The questionnaires were handed to 155 Davidoff Center staff members, of whom 107 (69%) filled them out with <20% missing data. The reliability of the questionnaire was assessed using the Cronbach's alpha test (α = 0.789; corrected, α = 0.82).

Of the 107 respondents, 85% were Jewish, 8% Muslim, and 7% other. 74% were Israeli-born, and 60% had ≥10 years of oncology experience. The respondents included 37 (35%) physicians (22 oncology, 15 hemato-oncology), 49 (46%) nurses (36 oncology, 13 hemato-oncology), 8 (7%) social workers, 4 (4%) psychologists, and 9 (8%) that were unspecified (Table 1).

**Table 1:** Participant's characteristics (n = 107).

Median age, years		41–45
Gender, n (%)		
	Male	25 (23)
	Female	76 (71)
	Unspecified	6 (6)
Marital status, n (%)		
	Single	14 (13)
	Married	77 (72)
	Widower	2 (2)
	Divorced	7 (7)
	Other	1 (1)
	Unspecified	6 (6)
Born, n (%)		
	Israel	79 (74)
	Former Soviet Union	6 (6)
	Europe	6 (6)
	USA	2 (2)
	North Africa	1 (1)
	Other	6 (6)
	Unspecified	7 (7)
Self-defined religion, n (%)		
	Jewish	91 (85)
	Muslim	9 (8)
	Christian	1 (1)
	Unspecified	6 (6)
Position, n (%)		
	Physician	37 (35)
	Nurse	49 (46)
	Social Worker	8 (7)
	Psychologist	4 (4)
	Unspecified	9 (8)
Department, n (%)		
	Oncology	70 (65)
	Hemato-oncology	33 (31)
	Onco-psychology	4 (4)

Most respondents thought it was important to contact family members of deceased patients (73%), that such interaction was also important to staff members (66%), that it provided closure to the staff members (79%), and that it was professionally appropriate (84%). Forty-one percent of respondents indicated that they contact more than 50% of families. Contacting bereaved families was considered to be within the responsibility of the treating physician (90%), nurses (84%), or social workers (89%). The preferable means of communication was a phone call (88%), followed by a condolence letter (75%). The main barriers to contacting bereaved families included emotional overload (68%) and lack of time (63%). More than half of respondents (60%) indicated they would like to have additional communication tools with respect to contacting bereaved families. In a multivariate analysis, age, gender, country of birth, marital status, number of children, years of oncology experience, and number of dying patients treated by the oncology professional, were mostly non-significant variables, whereas the position (physician or nurse) and primary workplace (outpatient setting vs. other settings) were significant variables both to respondents’ perspectives regarding the importance of contacting bereaved families, and to actually contacting them (Table 2).

**Table 2:** Responses by position and work setting (n = 107).

		Proportion of respondents agreeing with the statement						
		All	By position			By work setting		
	Statement	n/n (%)	Physicians, n/n (%)	Nurses, n/n (%)	*p*	Outpatient setting n/n (%)	Other setting n/n (%)	*p*
1	**I think it is important to contact grieving families**	77/106 (73)	33/37 (89)	25/48 (52)	**0.0001**	40/43 (93)	30/53 (57)	**<0.0001**
2	**Contacting grieving families is important to the family**	92/104 (88)	37/37 (100)	36/47 (77)	**0.002**	43/43 (100)	41/52 (79)	**0.001**
3	**Contacting grieving families is important to the caregiver (staff member)**	69/104 (66)	30/37 (81)	21/44 (48)	**0.001**	37/43 (86)	26/52 (50)	**0.0001**
4	**If I contact a grieving family**^**a**^							
	It gives me closure, as a caregiver	72/91 (79)	29/35 (83)	27/40 (68)	0.127	35/40 (88)	30/44 (68)	**0.035**
	I am acting professionally	76/91 (84)	32/35 (91)	25/37 (68)	**0.013**	37/40 (93)	32/43 (74)	**0.028**
	It is according to institutional guidelines	31/68 (46)	17/29 (59)	9/28 (32)	**0.045**	16/32 (50)	13/31 (42)	0.521
	It is an opportunity to say good bye to the family	77/91 (85)	31/34 (91)	30/41 (73)	**0.046**	36/39 (92)	34/45 (76)	**0.04**
5	**I think all grieving families should be contacted**	45/105 (43)	21/37 (57)	14/47 (30)	**0.013**	26/43 (60)	15/52 (29)	**0.002**
6	**If a grieving family is contacted, it should be done by the following staff member**^**a**^							
	Treating physician	83/92 (90)	34/34 (100)	34/41 (83)	**0.011**	37/38 (97)	38/44 (86)	0.075
	Nurse	63/75 (84)	22/23 (96)	29/37 (78)	0.068	27/29 (93)	32/39 (82)	0.183
	Social worker	74/83 (89)	23/24 (96)	37/43 (86)	0.209	27/29 (93)	41/46 (89)	0.565
7	**I prefer not to reveal my feelings in front of a grieving family**	36/103 (35)	10/36 (28)	18/47 (38)	0.315	11/41 (27)	21/52 (40)	0.172
8	**I initiate a meeting with grieving family members**	21/98 (21)	25/32 (78)	6/45 (13)	0.324	11/40 (27)	9/50 (18)	0.281
9	**If members of a grieving family request to meet me, I agree**	90/101 (89)	37/37 (100)	34/44 (77)	**0.002**	43/43 (100)	38/49 (78)	**0.001**
10	**I try to view the patient and his family as one unit**	97/106 (92)	32/37 (86)	6/42 (14)	0.243	40/43 (93)	49/54 (91)	0.685
11	**I contact all grieving families of patients that I treated**	33/97 (34)	19/36 (53)	6/42 (14)	**≤0.0001**	24/42 (57)	7/48 (15)	**0.0001**
12	**I contact >50% of grieving families of patients that I treated**	37/90 (41)	23/30 (77)	8/42 (19)	**≤0.0001**	25/35 (71)	9/47 (19)	**0.0001**
13	**I contact <50% of grieving families of patients that I treated**	34/81(42)	10/23 (43)	14/39 (36)	0.554	11/31 (35)	18/42 (43)	0.525
14	**I hardly contact grieving families of patients that I treated**	39/85 (46)	4/27 (15)	31/40 (78)	**≤0.0001**	2/33 (6)	32/45 (71)	**0.0001**
15	**I think that the preferable way to contact a grieving family is by**^**a**^							
	A phone call	77/88 (88)	33/34 (97)	26/35 (74)	**0.007**	38/39 (97)	34/43 (79)	**0.011**
	A home visit	30/75 (40)	8/29 (28)	16/29 (55)	**0.033**	8/31 (26)	20/38 (53)	**0.024**
	A letter	64/85 (75)	25/30 (83)	24/37 (65)	0.09	32/39 (82)	27/39 (69)	0.187
16	**Things preventing me from contacting grieving families**^**a**^							
	Emotional overload	52/76 (68)	16/24 (67)	27/35 (77)	0.374	15/30 (50)	33/40 (83)	**0.004**
	Lack of time	50/79 (63)	17/24 (71)	20/36 (56)	0.233	19/32 (59)	26/41 (63)	0.725
	I do not think it is important enough	10/69 (14)	2/22 (9)	6/30 (20)	0.281	2/30 (7)	6/34 (18)	0.185
	I do not have the appropriate tools	26/68 (38)	3/20 (15)	19/32 (59)	**0.002**	3/28 (11)	21/36 (58)	**0.0001**
17	**The period of time I have cared for a patient affects my decision whether to contact the family after his/her death**	74/101 (73)	31/36 (86)	31/47 (66)	0.316	34/42 (81)	36/52 (69)	0.195
	The longer the relationship, the more important it is for me to contact the family after the patient’s death	80/101 (79)	33/36 (92)	30/46 (65)	**0.005**	38/42 (90)	37/51 (73)	**0.029**
18	**The age of the patient is a factor that influences contacting his/her grieving family**	29/101 (29)	11/36 (31)	12/46 (26)	0.655	11/42 (26)	16/51 (31)	0.584
	It is more important for me to contact grieving families of younger patients	37/102 (36)	18/36 (50)	14/48 (29)	0.052	17/42 (40)	18/52 (35)	0.559
19	**I would like to acquire more tools for coping and contacting grieving families**	59/98 (60)	16/33 (49)	32/47 (68)	0.078	20/39 (51)	36/52 (69)	0.082
20	**For those who write letters: writing letters to grieving families is important to me because**^**a**^							
	It gives me closure, as a caregiver	24/34 (71)	12/14 (86)	6/12 (50)	**0.049**	15/18 (83)	7/13 (54)	0.074
	It is the right thing to do, professionally	27/36 (75)	12/14 (86)	7/13 (54)	0.07	16/19 (84)	9/14 (64)	0.187
	It is according to institutional guidelines	14/27 (52)	8/12 (67)	3/10 (30)	0.087	8/14 (57)	5/11 (45)	0.561
	It is an opportunity to say good bye to the family	26/35 (74)	12/14 (86)	7/12 (58)	0.117	16/19 (84)	8/13 (62)	0.146

^**a**^Respondents were instructed to respond to all sub-statements.

### Impact of oncology profession

Physicians and nurses had significantly different perspectives with respect to their role in bereavement follow-up (Table 2), with more physicians than nurses agreeing that it was important to contact grieving families (89 vs. 52%, *p* = 0.0001), and that such contact was important to the grieving families (100 vs. 77%, *p* = 0.002), as well as to the caregiver (81 vs. 48%, *p* = 0.001). In addition, more physicians than nurses thought that all grieving families should be contacted (57 vs. 30%, *p* = 0.013), and that this contact should be made by the treating physician (100 vs. 83%, *p* = 0.011). Respondents indicated that the longer the relationship with the patient, the more important it was for them to contact the family after the patient’s death (92 vs. 65%, *p* = 0.005). In contrast, more nurses than physicians indicated that lack of appropriate tools prevented them from contacting bereaved families (59 vs. 15%, *p* = 0.002).

### Impact of the primary workplace

Forty-seven respondents (44%) indicated that the outpatient clinic was their primary workplace. These respondents differed significantly from the other respondents (Table 2) in that more of them stated that it was important to contact grieving families (93 vs. 57%, *p*<0.0001), and that such contact is important to the grieving families (100 vs. 79%, *p* = 0.001) as well as to the caregiver (86 vs. 50%, *p* = 0.0001), to whom it provides closure (88 vs. 68%, *p* = 0.035). In addition, more respondents working in the outpatient clinic indicated that they contacted all grieving families of patients they treat (57 vs. 15%, *p* = 0.0001) and that the longer the relationship with the patients the more important it was to them to contact the family after the patient’s death (90 vs. 73%, *p* = 0.029). Statistically significant was the finding that more respondents working in settings outside the outpatient clinic indicated that a lack of appropriate tools and emotional overload prevented them from contacting bereaved families (58 vs. 11%, *p* = 0.0001, 83 vs. 50%, *p* = 0.004 respectively).

These observed differences prompted us to explore the differences between nurses working in the outpatient clinic and those working in other settings (Table 3). We found that the perspectives of nurses working in the outpatient clinic differed significantly from those working in other settings, and were similar to those of physicians working in the outpatient setting. Thus, more nurses working in the outpatient clinic than in other settings agreed that contacting grieving families is important to the caregiver (83 vs. 38%, *p* = 0.039), and more of them indicated that they contacted all grieving families (60 vs. 8%, *p* = 0.002). Furthermore, nurses working in other settings than the outpatient clinic were more likely to agree that emotional overload prevented them from contacting grieving families (83 vs. 40%, *p* = 0.037).

**Table 3:** Responses of nurses by work setting (n = 49).

		Proportion of respondents agreeing with the statement		
		Outpatient setting, n/n (%)	Other setting, n/n (%)	*p*
1	**I think it is important to contact grieving families**	5/6 (83)	19/40 (48)	0.101
2	**Contacting grieving families is important to the family**	6/6 (100)	28/39 (72)	0.134
3	**Contacting grieving families is important to the caregiver (staff member)**	5/6 (83)	15/39 (38)	**0.039**
4	**If I contact a grieving family**^**a**^			
	It gives me closure, as a caregiver	5/6 (83)	21/33 (64)	0.346
	I am acting professionally	5/6 (83)	20/30 (67)	0.418
	It is according to institutional guidelines	3/5 (60)	6/22 (27)	0.161
	It is an opportunity to say good bye to the family	5/5 (100)	23/34 (68)	0.133
5	**I think all grieving families should be contacted**	3/6 (50)	11/39 (28)	0.283
6	**If a grieving family is contacted, it should be done by the following staff member**^**a**^			
	Treating physician	5/6 (83)	27/33 (82)	0.929
	Nurse	5/6 (83)	23/30 (77)	0.72
	Social worker	5/6 (83)	30/34 (88)	0.879
7	**I prefer not to reveal my feelings in front of a grieving family**	2/6 (33)	16/39 (41)	0.72
8	**I initiate a meeting with grieving family members**	2/6 (33)	4/38 (11)	0.13
9	**If members of a grieving family request to meet me, I agree**	6/6 (100)	26/36 (72)	0.139
10	**I try to view the patient and his family as one unit**	6/6 (100)	38/41 (93)	0.493
11	**I contact all grieving families of patients that I treated**	3/5 (60)	3/36 (8)	**0.002**
12	**I contact >50% of grieving families of patients that I treated**	3/5 (60)	4/36 (11)	**0.006**
13	**I contact <50% of grieving families of patients that I treated**	2/6 (33)	11/32 (34)	0.961
14	**I hardly contact grieving families of patients that I treated**	0/5 (0)	30/34 (88)	**<0.0001**
15	**I think that the preferable way to contact a grieving family is by**^**a**^			
	A phone call	3/4 (75)	22/30 (73)	0.943
	A home visit	0/2 (0)	16/27 (59)	0.104
	A letter	5/6 (83)	18/29 (62)	0.318
16	**Things preventing me from contacting grieving families**^**a**^			
	Emotional overload	2/5 (40)	24/29 (83)	**0.037**
	Lack of time	3/6 (50)	17/30 (57)	0.764
	I do not think it is important enough	0/5 (0)	6/25 (24)	**0.0221**
	I do not have the appropriate tools	1/5 (20)	18/27 (67)	0.051
17	**The period of time I have cared for a patient affects my decision whether to contact the family after his/her death**	5/6 (83)	26/39 (67)	0.412
	The longer the relationship, the more important it is for me to contact the family after the patient’s death	5/6 (83)	25/38 (66)	0.391
18	**The age of the patient is a factor that influences contacting his/her grieving family**	3/6 (50)	9/38 (24)	0.179
	It is more important for me to contact grieving families of younger patients	3/6 (50)	11/40 (28)	0.264
19	**I would like to acquire more tools for coping and contacting grieving families**	3/6 (50)	27/40 (68)	0.432
20	**For those who write letters: writing letters to grieving families is important to me because**^**a**^			
	It gives me closure, as a caregiver	3/4 (75)	3/8 (38)	0.221
	It is the right thing to do, professionally	3/4 (75)	4/9 (44)	0.308
	It is according to institutional guidelines	2/3 (67)	6/7 (86)	0.098
	It is an opportunity to say good bye to the family	3/4 (75)	4/8 (50)	0.408

^**a**^Respondents were instructed to respond to all sub-statements.

### The impact of self-defined religion

Respondents identified as Muslim agreed less than others that contacting grieving families is important to the family (56 vs. 92%, *p* = 0.001), and to the caregiver (22 vs. 70%, *p* = 0.003), that such contact provides an opportunity to say goodbye to the grieving family (40 vs. 87%, *p* = 0.004), and that the preferable way for such contact is by phone (57 vs. 90%, *p* = 0.011).

## Discussion

This study is unique, since it investigated interactions of an interdisciplinary cancer care team with families of deceased patients. Differences were found between the various oncology professionals (physicians vs. nurses), between those working in an outpatient setting vs. other work settings, and between professionals of different self-defined religions. The study also showed that most staff members consider contacting bereaved families to be important, and that most of them thought they lacked proper tools to deal with such interaction. Variables such as age, gender, or a high number of deceased patients did not affect the willingness of staff members to contact bereaved families, unlike previously-reported findings [8–10]. Our study showed that contacting bereaved families was more important to physicians than to nurses (except for nurses working in the outpatient clinic). This difference probably stems from physicians’ perception of themselves as the main, constant caregivers, making them feel obligated to the patient’s family, as well as from the fact that some of the nurses only interact with the patients for short periods of time, and may not even be aware of their death [9]. Notably, this finding contradicts findings from a 2010 Israeli study, in which 42% of surveyed physicians (medical/radiation oncologists) said that contacting bereaved families was beyond their professional duty [11]. Regarding nurses working in outpatient clinics, our findings are consistent with reports demonstrating differences between nurses working in the outpatient vs. the inpatient setting, and suggesting that devotion of nurses to patients and their families may increase by strengthening the relationships between physicians and nurses, addressing nurses' workloads, and implementing communication-strengthening strategies [12, 13].

Cancer care approaches have evolved to be patient-centered, with an interdisciplinary health-care team (physicians, nurses, social workers, and psychologists) attending to the patients and/or their families and providing continuity of care (in the outpatient setting) [14–19]; this might explain the observed differences in perspectives regarding bereavement follow-up between staff members working in the outpatient setting and other settings. As revealed herein, the differences between physicians and nurses seem to stem from the workplace and the nature of the interaction with patients rather than from their role *per se*, as the perspectives of nurses in the outpatient clinic were similar to those of physicians.

Staff at the out patients' setting, agreed less than others that emotional overload prevents them from contacting bereaved families. Although the literature does address the issue of emotional overload among oncology health care providers and its consequence regarding behaviors [20, 21], the contribution of differences in primary workplace has not been highlighted until now.

To the best of our knowledge, our study is the first to demonstrate the impact of cultural differences on perspectives surrounding bereavement follow-up, although the differences in positions (the majority of Muslims were oncology nurses) may account for some of the observed difference. In addition, the small number of Muslim participants in our study limits our ability to draw conclusions. Published reports addressing cultural differences focused on patients’ cultural differences and how they affect the interactions with the medical staff, but not on cultural variability among staff, possibly due to the assumption that medical staff members adopt the expected professional code without letting their cultural background get in the way. Our findings regarding cultural or religious differences raise the question of staff members' cultural or religious variability and its effects on professional decision making. However, Campinha-Baccot and Granot & Pollak suggested that when caregivers acknowledge, understand and reflect on their own values and cultures, they better understand and address patients’ cultural variability and needs [22, 23].

In addition, our study also revealed oncology staff members' need for tools to support them in contacting bereaved families and gaining closure on their patients’ death. This could be achieved through professional development programs (e.g., education, mentoring). Regarding the fact that only half of the participants agreed with the claim: 'Writing letters is important due to institutional guidelines', in this matter, there is no reinforcement to health care providers. This remains as an educational process to be continued.

The study is limited by its descriptive cross-sectional design and a relatively small sample of workers in a single tertiary center, limiting our ability to draw definite conclusions regarding certain subgroups (e.g., nurses working in the outpatient setting or cultural/religious differences).

In conclusion, this study investigating the interactions between oncology staff members and bereaved families in Israel demonstrated differences in perspectives between physicians and nurses, between staff members working in the outpatient and other settings, and between oncology staff of different self-defined religions.

## Supporting Information

S1 File FACPQ—Factors Affecting Communication Patterns Questionnaire. (DOCX)
