# Supplementary material for: Factors Affecting Communication Patterns between Oncology Staff and Family Members of Deceased Patients: A Cross-Sectional Study
Source: PLoS One. 2016 Sep 28;11(9):e0162813. doi: 10.1371/journal.pone.0162813 (PMC5040255; doi:10.1371/journal.pone.0162813)
Supplement: S1 File — (DOCX) [file pone.0162813.s001.docx]

| **5**  **Do not agree at all** | **4** | **3** | **2** | **1**  **Agree very much** | **Statements** |
| --- | --- | --- | --- | --- | --- |
|  |  |  |  |  | \| 1 \| **I think it is important to contact grieving families** \|  \|  \|  \|  \|  \| 30/53 (57) \| **<0.0001** \| \| --- \| --- \| --- \| --- \| --- \| --- \| --- \| --- \| --- \| \| 2 \| **Contacting grieving families is important to the family** \|  \|  \|  \|  \|  \| 41/52 (79) \| **0.001** \| \| 3 \| **Contacting grieving families is important to the caregiver (staff member)** \|  \|  \|  \|  \|  \| 26/52 (50) \| **0.0001** \| \| 4 \| **If I contact a grieving family** \|  \|  \|  \|  \|  \|  \|  \| \|  \| It gives me closure, as a caregiver \|  \|  \|  \|  \|  \| 30/44 (68) \| **0.035** \| \|  \| I am acting professionally \|  \|  \|  \|  \|  \| 32/43 (74) \| **0.028** \| \|  \| It is according to institutional guidelines \|  \|  \|  \|  \|  \| 13/31 (42) \| 0.521 \| \|  \| It is an opportunity to say good bye to the family \|  \|  \|  \|  \|  \| 34/45 (76) \| **0.04** \| \| 5 \| **I think all grieving families should be contacted** \|  \|  \|  \|  \|  \| 15/52 (29) \| **0.002** \| \| 6 \| **If a grieving family is contacted, it should be done by the following staff member** \|  \|  \|  \|  \|  \|  \|  \| \|  \| Treating physician \|  \|  \|  \|  \|  \| 38/44 (86) \| 0.075 \| \|  \| Nurse \|  \|  \|  \|  \| 27/29 (93) \| 32/39 (82) \| 0.183 \| \|  \| Social worker \|  \|  \|  \|  \| 27/29 (93) \| 41/46 (89) \| 0.565 \| \| 7 \| **I prefer not to reveal my feelings in front of a grieving family** \|  \|  \|  \|  \| 11/41 (27) \| 21/52 (40) \| 0.172 \| \| 8 \| **I initiate a meeting with grieving family members** \|  \|  \|  \|  \| 11/40 (27) \| 9/50 (18) \| 0.281 \| \| 9 \| **If members of a grieving family request to meet me, I agree** \|  \|  \|  \|  \| 43/43 (100) \| 38/49 (78) \| **0.001** \| \| 10 \| **I try to view the patient and his family as one unit** \|  \|  \|  \|  \| 40/43 (93) \| 49/54 (91) \| 0.685 \| \| 11 \| **I contact all grieving families of patients that I treated** \|  \|  \|  \|  \| 24/42 (57) \| 7/48 (15) \| **0.0001** \| \| 12 \| **I contact >50% of grieving families of patients that I treated** \|  \|  \|  \|  \| 25/35 (71) \| 9/47 (19) \| **0.0001** \|  \| 13 \| **I contact <50% of grieving families of patients that I treated** \|  \|  \|  \|  \| 11/31 (35) \| 18/42 (43) \| 0.525 \| \| --- \| --- \| --- \| --- \| --- \| --- \| --- \| --- \| --- \| \| 14 \| **I hardly contact grieving families of patients that I treated** \|  \|  \|  \|  \| 2/33 (6) \| 32/45 (71) \| **0.0001** \| \| 15 \| **I think that the preferable way to contact a grieving family is by** \|  \|  \|  \|  \|  \|  \|  \| \|  \| A phone call \|  \|  \|  \|  \| 38/39 (97) \| 34/43 (79) \| **0.011** \| \|  \| A home visit \|  \|  \|  \|  \| 8/31 (26) \| 20/38 (53) \| **0.024** \| \|  \| A letter \|  \|  \|  \|  \| 32/39 (82) \| 27/39 (69) \| 0.187 \| \| 16 \| **Things preventing me from contacting grieving families** \|  \|  \|  \|  \|  \|  \|  \| \|  \| Emotional overload \|  \|  \|  \|  \| 15/30 (50) \| 33/40 (83) \| **0.004** \| \|  \| Lack of time \|  \|  \|  \|  \| 19/32 (59) \| 26/41 (63) \| 0.725 \| \|  \| I do not think it is important enough \|  \|  \|  \|  \| 2/30 (7) \| 6/34 (18) \| 0.185 \| \|  \| I do not have the appropriate tools \|  \|  \|  \|  \| 3/28 (11) \| 21/36 (58) \| **0.0001** \| \| 17 \| **The period of time I have cared for a patient affects my decision whether to contact the family after his/her death** \|  \|  \|  \|  \| 34/42 (81) \| 36/52 (69) \| 0.195 \| \|  \| The longer the relationship, the more important it is for me to contact the family after the patient’s death \|  \|  \|  \|  \| 38/42 (90) \| 37/51 (73) \| **0.029** \| \| 18 \| **The age of the patient is a factor that influences contacting his/her grieving family** \|  \|  \|  \|  \| 11/42 (26) \| 16/51 (31) \| 0.584 \| \|  \| It is more important for me to contact grieving families of younger patients \|  \|  \|  \|  \| 17/42 (40) \| 18/52 (35) \| 0.559 \| \| 19 \| **I would like to acquire more tools for coping and contacting grieving families** \|  \|  \|  \|  \| 20/39 (51) \| 36/52 (69) \| 0.082 \| \| 20 \| **For those who write letters: writing letters to grieving families is important to me because** \|  \|  \|  \|  \|  \|  \|  \| \|  \| It gives me closure, as a caregiver \|  \|  \|  \|  \| 15/18 (83) \| 7/13 (54) \| 0.074 \| \|  \| It is the right thing to do, professionally \|  \|  \|  \|  \| 16/19 (84) \| 9/14 (64) \| 0.187 \| \|  \| It is according to institutional guidelines \|  \|  \|  \|  \| 8/14 (57) \| 5/11 (45) \| 0.561 \| \|  \| It is an opportunity to say good bye to the family \|  \|  \|  \|  \| 16/19 (84) \| 8/13 (62) \| 0.146 \| |
